# ADHESION TO TREATMENT BY CHILDREN WITH CONGENITAL HYPOTHYROIDISM: KNOWLEDGE OF CAREGIVERS IN BAHIA STATE, BRAZIL

**DOI:** 10.1590/1984-0462/2021/39/2020074

**Published:** 2021-04-02

**Authors:** Lara Novais Santos Brito, Caio Leônidas Oliveira de Andrade, Crésio de Aragão Dantas Alves

**Affiliations:** a Escola Bahiana de Medicina e Saúde Pública, Salvador, BA, Brazil.; b Universidade do Estado da Bahia, Salvador, BA, Brazil.; c Universidade Federal da Bahia, Salvador, BA, Brazil.

**Keywords:** Congenital hypothyroidism, Caregivers, Cooperation and adherence to treatment, Comprehensive health care, Hipotireoidismo congênito, Cuidadores, Cooperação e adesão ao tratamento, Assistência integral à saúde

## Abstract

**Objective:**

To investigate knowledge of caregivers of children with congenital hypothyroidism (CH), followed in a public reference service, as well as their associations with treatment adherence.

**Methods:**

Exploratory, descriptive, cross-sectional study with convenience sample. Medical records of 158 patients diagnosed with congenital hypothyroidism were analyzed, and data were evaluated by applying a previously prepared questionnaire to caregivers from 2014 to 2016. Statistical analysis used the chi-square and the Spearman’s correlation tests, being significant p-value ≤0.05.

**Results:**

Females were predominant among caregivers (94.3%), with a mean age of 31 years, from inland cities (77.8%). There was a predominance of socioeconomic class C (59.5%) and incomplete primary education (35.7%). More than half of patients (53.2%) with CH had an adequate hormonal control. Approximately one third of caregivers had poor knowledge (37.3%) or was unaware (24.1%) about the meaning of congenital hypothyroidism. The low knowledge level of the disease was observed to be related to caregivers’ educational level (p=0.004).

**Conclusions:**

Lack of education of caregivers was a barrier to be faced when monitoring children with CH. This reality requires greater attention from health professionals to ensure that they use clear language when giving instructions to caregivers, and that caregivers have adequately understood the proposed recommendations.

## RESUMO

**Objetivo:**

Investigar o conhecimento dos cuidadores das crianças com hipotireoidismo congênito (HC) acompanhadas em um serviço público de referência, bem como as suas associações com a adesão ao tratamento.

**Métodos:**

Trata-se de um estudo exploratório, descritivo, de corte transversal, com amostra de conveniência. Foram analisados os prontuários de 158 pacientes com diagnóstico de HC e avaliados os dados obtidos pela aplicação de um questionário previamente elaborado, destinado aos cuidadores, durante o período de 2014 a 2016. A análise estatística foi feita com o teste do qui-quadrado e a Correlação de Spearman, para correlacionar a pontuação do questionário, sendo significante o valor de p≤0,05.

**Resultados:**

Os cuidadores eram predominantemente do sexo feminino (94,3%), com média de idade de 31 anos, proveniente de cidades do interior (77,8%). Verificou-se predomínio da classe socioeconômica C (59,5%) e ensino fundamental incompleto (35,7%) na amostra dos cuidadores. Metade dos pacientes (53,2%) apresentava controle hormonal adequado. Aproximadamente 1/3 dos cuidadores demonstraram conhecimento ruim (37,3%) ou até mesmo desconheciam (24,1%) o HC. Observou-se que o baixo nível de conhecimento da doença estava diretamente relacionado com o nível educacional dos cuidadores (p=0,004).

**Conclusões:**

Os achados sugerem que a falta de instrução dos cuidadores é uma barreira a ser enfrentada durante o acompanhamento de crianças com HC, o que requer uma maior atenção do profissional de saúde, para garantir uma linguagem clara e um entendimento adequado das recomendações propostas.

## INTRODUCTION

Congenital hypothyroidism (CH) is a chronic endocrine disturbance, affecting one in every 2,000 to 4,000 newborns.^1^ ^,^ ^2^ In Brazil, prevalence is similar to worldwide parameters, ranging between 1:2595 to 1:4795 live newborns.^3^ ^,^ ^4^

CH is characterized by the decline in thyroid hormone (TH) production, mainly due to defects in gland formation, thyroid dysgenesis.^5^ ^,^ ^6^ This disturbance results in a generalized reduction in metabolic processes, which interfere in the neurological and motor growth, and development of children.^6^

CH is traced by neonatal screening tests, due to their elevated sensitivity and efficacy. The recommended period for collecting blood sample is between the 3^rd^ and 5^th^ day of life.^7^ The presence of any hormonal imbalance must be proved with confirmatory tests, which must be performed as early as possible, ideally between the first and second weeks of the newborn’s life.

Continuous TH replacement may revert metabolic changes of the disease, thus avoiding irreversible damage to the central nervous system. However, for therapy to be successful, early diagnosis and immediate therapeutic intervention are needed.^6^ ^,^ ^8^

Although CH is one of the most preventable causes of mental retardation, lack of knowledge of caregivers about the importance of having the heel prick test performed, in addition to the benefits of adequately following treatment, is a barrier to the clinical improvement of these patients. Furthermore, the absence or non-specificity of symptoms in the initial stage of the disease lead to difficulty in making the clinical diagnosis. In general, symptoms usually appear when neurological damages are almost irreversible.^5^ Seen that, determining the knowledge level of parents and/or caregivers about CH is extremely important, with the purpose of helping health professionals to detect the aspects related to low adherence and implement health promotion actions.

In view of that, the aim of the present study was to investigate the knowledge of caregivers about the treatment of children with CH, followed-up at a public reference service, to verify whether there is any association between metabolic control, sociodemographic data, and the knowledge level of caregivers of the disease.

## METHOD

Exploratory, descriptive, cross-sectional study with convenience sample and quantitative approach. Data were collected at the Neonatal Screening Reference Service (*Serviço de Referência de Triagem Neonatal* - NSRS) of Bahia State, from 2014 to 2016.

Records of patients with CH, periodically followed up at the NSRS, were analyzed. Patients under 18 had to sign the Free and Informed Agreement Form (FIAF). All those responsible for the children signed the Free and Informed Consent Form (FICF). Patients with central hypothyroidism, acquired hypothyroidism, with neurological and psychological abnormalities, or any other metabolic diseases were excluded from the study.

The present study was analyzed by the Research Ethics Committee (REC) of the participating institution, and approved by Report No. 534.704/2013, based on Resolution No. 466/12 of the Brazilian Health Council.

Parents and/or caregivers answered a structured questionnaire of closed questions, which contained subjective and specific questions about CH. Initially, interviewees had to provide caregivers’ own data (for example, age, sex, marital status, educational level, profession, relationship with the child), followed by questions about CH (for example, symptoms and treatment).

The questionnaire was elaborated before data collection began, with a prior study initially being conducted with a sample group of 10 individuals to verify the applicability of the study instrument regarding the level of difficulty found by participants when answering alternative responses and regarding the relevance of the questions asked. With this investigative method, the presence of incoherent factors, which could distort the study’s desired results, could be verified. Therefore, the first protocols applied were excluded from research. After revising incoherent items, a second study was applied to a new sample of 10 individuals. This new study showed absence of errors in the statements of the research instrument, that is, the structured questionnaire. Thus, sample of the second study was used to compose part of the sample of the present study, totaling 158 participants.

The questionnaire contained 12 questions: three of a subjective nature, which did not receive a score; and nine objective questions, which received scores. Each question was worth 0.5 to 2 points, according to their level of difficulty. The score acquired in the questionnaire was stratified into five categories of knowledge: excellent (9-10), good (6.8-8.9), regular (5-6.7), poor (3-4.9), and no knowledge (0-2.9).

For applying the protocols, parents/caregivers were submitted to a formal interview process. The technique of choice was the “face-to-face” modality, which consisted of oral questioning by the researcher, by reading out the items. After the due instructions were given, the interviewer had no further interference, because the sample had heterogeneous characteristics relative to age and educational level.

Adhesion to therapy was evaluated by analyzing the last three TSH values. Metabolic control was categorized into three classes: 1) adequate control (TSH between 0.5-15 µUI/mL); 2) hypotreatment (TSH above 15 µUI/mL); and 3) hypertreatment (TSH below 0.5 µUI/mL).^9^ ^,^ ^10^ The individuals who proceeded with three or more episodes of serum levels of TSH <0.5 µUI/mL or >15 µUI/mL, during the period of hormone follow-up, were considered having irregular serum hormone levels, and were classified as patients who were “hypertreated” and “hypotreated”, respectively.^10^

Socioeconomic classification was based on the Brazilian Economic Classification Criterion.^11^ A score is attributed to each asset the family owns, and the sum of scores defines each class into categories A, B, C, and D-E. This socioeconomic stratification criterion depends not only on variables such as family income, educational level, amount of different assets owned, access to public services, but also on the place in which homes are localized, and the number of adults and minors (under 18) in each family.

Variables related to the patient (age of neonatal screening, age of diagnosis confirmation, quantity of consultations attended, sex, and TSH dosage) and to the caregiver (place of origin, sex, profession, marital status, age, family relationship, educational level, and score obtained in the questionnaire to which the person was submitted).

In addition, medical data collected from the patients’ record charts were evaluated. For this purpose, the software *Statistical Package for the Social Sciences* (SPSS), version 21.0, was used. Categorical variables were expressed in absolute and relative values (percentages). Continuous variables with normal distribution were expressed in means and standard deviation, and those with asymmetric distribution, in medians and interquartile intervals.

The chi-square test was used for association among categorical variables, classification in the questionnaire, such as control of metabolism, and caregivers’ sociodemographic data. Spearman’s correlation was used to correlate the score obtained in the questionnaire. For all analyses, p≤0.05 was established.

## RESULTS

**Figure 1:**
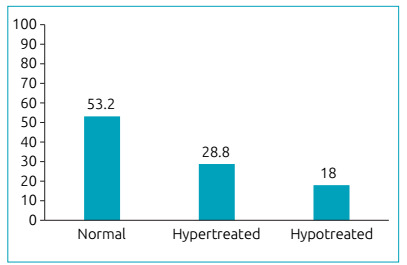
Percentage (%) of patients according to their serum thyroid-stimulating hormone level (n=158).

A total of 158 patients with congenital hypothyroidism were evaluated, ranging from one to seven years old. There was higher prevalence of the female sex (55.4%) and patients coming from the interior regions of the state (77.8%). Of the 158 caregivers, most were female (94.3%), with a mean age of 31 years (ranging between 22 and 40), belonging to the socioeconomic Class C (59.5%), with incomplete primary schooling (35.7%), and informal employment (65.4%) (Table 1). The heel prick test and diagnostics tests were performed between three and 104 days old, and 19 and 627 days old, respectively (Table 2). Approximately half of the patients evaluated had an adequate use of medication (53.2%), as illustrated in Figure 1.

**Table 1:** Sociodemographic data of caregivers of children with congenital hypothyroidism.

	n (%)
Sex	
Male	09 (5.7)
Female	149 (94.3)
Relationship	
Father	08 (5.1)
Mother	134 (84.8)
Brother/sister	04 (2.5)
Grandfather/grandmother	07 (4.4)
Uncle/aunt	04 (2.5)
Profession	
Formal work	54 (34.6)
Informal work	102 (65.4)
Educational level	
No formal education	03 (1.9)
Incomplete primary schooling	56 (35.7)
Complete primary schooling	34 (21.7)
Incomplete high schooling	13 (8.3)
Complete high schooling	42 (26.8)
Incomplete university education	07 (4.5)
Complete university education	02 (1.3)
Socioeconomic classification	
Class A	02 (1.3)
Class B	43 (27.2)
Class C	94 (59.5)
Class D/E	19 (12.0)

n: number of patients; SD: standard deviation.

**Table 2:** Clinical data of individuals with congenital hypothyroidism.

	Median (QI-QIII)
Age (years old)	4.0 (1.0-7.0)
Age at diagnosis (days)	40.5 (29.0-57.8)
Duration of disease (years)	3.1 (0.6-6.1)
Heel Prick Test	
TSH level (µUI/ml)	31.0 (13.4-147.4)
Age (days)	15.5 (8.0-24.8)
Confirmatory Exam	
TSH level (µUI/ml)	39.5 (11.1-101.0)
Age (days)	41.0 (30.0-54.5)
Number of consultations at the NSRS	13.0 (8.0-18.0)
**Classification at USG**	**n (%)**
Thyroid dyshormonogenesis	01 (0.6)
Thyroid dysgenesis	24 (15.2)
Normal	02 (1.3)
Not defined	131 (82.9)

n: number of participants; Q: quartile; TSH: thyroid-stimulating hormone; NSRS: Neonatal Screening Reference System *(Serviço de Referência de Triagem Neonatal)*; USG: ultrasonography; Not defined: child had not undergone the exam until the time of study.

**Figure 2:**
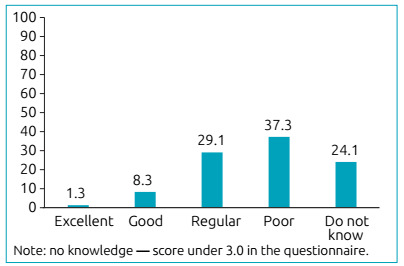
Distribution of the knowledge level of caregivers about congenital hypothyroidism (%).

Scores ranging from 3 to 5.3 were achieved in the questionnaire, with a mean value of 4.3. Moreover, this showed that most interviewees had a poor (37.3) to regular (29.1%) level of knowledge, or knew nothing (24.1%) about the disease, its characteristics, diagnosis, and therapy (Figure 2). The relation between the knowledge level of caregivers was also assessed in the questionnaire, with the classification of metabolic control of patients and their socioeconomic class (Table 3).

**Table 3:** Association of knowledge of caregivers of the disease, with sociodemographic data.

	Excellent	Good	Regular	Poor	Do not know	p-value
	n (%)	n (%)	n (%)	n (%)	n (%)	
Socioeconomic Classification						
Class A	00 (0.0)	00 (0.0)	01 (50.0)	01 (50.0)	00 (0.0)	
Class B	01 (2.3)	03 (7.0)	17 (39.5)	16 (37.2)	06 (14.0)	0.671
Class C	01 (1.1)	08 (8.5)	26 (27.7)	34 (36.2)	25 (26.6)	
Class D/E	00 (0.0)	02 (10.5)	02 (10.5)	08 (42.1)	07 (36.8)	
Category of Metabolic control						
Normal	00 (0.0)	04 (5.4)	23 (31.1)	35 (47.3)	12 (16.2)	
Hypertreated	01 (2.5)	04 (10.0)	12 (30.0)	12 (30.0)	11 (27.5)	0.465
Hypotreated	00 (0.0)	01 (4.0)	07 (28.0)	10 (40.0)	07 (28.0)	
Place of origin						
Capital	01 (3.3)	03 (10.0)	08 (26.7)	13 (43.3)	05 (16.7)	0.661
Interior	01 (01.0)	06 (5.7)	33 (31.4)	40 (38.1)	25 (23.8)	
Sex						
Male	00 (0.0)	01 (11.1)	03 (33.3)	02 (22.2)	03 (33.3)	0.878
Female	02 (1.3)	12 (8.1)	43 (28.9)	57 (38.3)	35 (23.5)	
Occupation						
Formal	01 (1.9)	09 (16.7)	14 (25.9)	21 (38.9)	09 (16.7)	0.059
Informal	01 (1.0)	04 (3.9)	31 (30.4)	38 (37.3)	28 (27.5)	
Educational level						
No formal education	00 (00.0)	00 (0.0)	00 (0.0)	01 (33.3)	02 (66.7)	
Incomplete primary schooling	01 (1.8)	01 (1.8)	10 (17.9)	26 (46.4)	18 (32.1)	
Complete primary schooling	00 (0.0)	02 (5.9)	12 (35.3)	10 (29.4)	10 (29.4)	
Incomplete high schooling	01 (7.7)	00 (0.0)	03 (23.1)	05 (38.5)	04 (30.8)	*0.004
Complete high schooling	00 (0.0)	07 (16.7)	18 (42.9)	14 (33.3)	03 (7.1)	
Incomplete university education	00 (0.0)	00 (0.0)	00 (0.0)	01 (50.0)	01 (50.0)	
Complete university education	00 (0.0)	03 (42.9)	03 (42.9)	01 (14.3)	00 (0.0)	

n: number; %: percentage of p-value in chi-square test; *p<0.05.

## DISCUSSION

A predominance of the female sex over the male sex (1.2:1) could be observed in patients with CH - a little lower than the value found in literature (2:1).^12^ The female sex was prevalent not only among children, but also among their caregivers. This predominance of care reflects a social ideology of the female sex being linked to the responsibility for caring for their children - the so-called “maternal concern”.^13^

Most patients came from interior regions of the state, which demonstrated the absence of centers specialized in the follow-up of children with CH in these regions. This led to many families frequently having to travel from their cities to the capital, often by transport offered by the municipality, in search of specialized medical care.

Higher prevalence of the socioeconomic class C was observed in the studied sample, a fact that could be related to the phenomenon of rise in social status of this class in Brazil over the last few years. This is a result of greater social mobility, more access to education, occupation and income, information, technologies, and sanitary actions in the public and/or private network. These changes may have contributed to a higher level of knowledge of families about primary health care programs, which could explain their prevalence.^14^

Low participation of class A, followed by classes D-E, was observed. This low rate of individuals from socioeconomic class A suggests that they use other services, either via health insurance plans or care provided by their private network. This fact limited the analysis of level of knowledge of caregivers about the research topic, who formed part of this socioeconomic class, because their representation was non-significant. On the other hand, the low presence of class D-E could be related to the fact that most of their members had risen to class C, a social phenomenon visible after some changes in public policies in Brazil over the last few years.

The time in which neonatal screening and confirmation of diagnosis occurred was longer than the time recommended in literature, ranging between eight to 24 days, and 29 to 57 days, respectively. This fact was not ideal for starting treatment, because the age recommended for performing neonatal screening is from the 3^rd^ and 5^th^ day of life, whereas the confirmation of diagnosis and beginning of treatment must occur up to the 28^th^ day of life.^7^ However, results like those were observed in studies conducted in Brazil in the states of Rio de Janeiro,^15^ Mato Grosso,^16^ Sergipe,^17^ Tocantins,^18^ and Ceará.^19^ This delay in performing neonatal screening and confirmatory exams, shown by the present study and in other Brazilian states, probably resulted from the lack of knowledge of caregivers as to the method of screening and its importance, or due to the difficulty healthcare providers have in evaluating the test within the recommended time frame.^14^ ^,^ ^15^ ^,^ ^16^ ^,^ ^17^ ^,^ ^18^ ^,^ ^19^

Such delay could significantly interfere in the overall development of children with CH. Children who do not receive early treatment for CH may suffer significant harm to their neuro-psychomotor development, besides their cognitive and behavioral functions.^13^ This reality leads to an increasing cause for concern in cases of severe congenital hypothyroidism (agenesis and thyroid dyshormonogenesis), because when these conditions are not diagnosed and treated in a timely manner, they lead to irreversible damage to the central nervous system.^20^ Its prognosis mainly depends on the moment in which treatment begins and on the maintenance of adequate hormone control.^21^

In the present sample, only half of patients had an adequate hormone control, which means medication was being correctly administered. All caregivers must repeatedly be reminded about the use of medication. Considering this, it was possible that, in some children with an inadequate hormone control, lack of care may have occurred during administration of hormone therapy, either due to the use of an excessive dose, or the time of administering medication was forgotten, or even because of caregivers’ difficulty in understanding the explanations provided by professionals. Studies also point out that caregivers may affirm they had correctly followed the doctor’s instructions during consultation, because they were faced with a figure such as the doctor, who represented authority.^22^ ^,^ ^23^ This situation would compromise treatment efficiency, because caregivers maybe cannot analyze the consequences associated to their acts of following or not following the doctor’s instructions.^4^ ^,^ ^22^ ^,^ ^24^ Thus, inappropriate thyroid hormone replacement therapy in children with CH could result in irreversible sequela to their body.^25^

Adherence corresponds to understanding the costs and benefits of following the therapeutic plan suggested by the health professional.^26^ In the case of chronic diseases such as CH, understanding adhesion extends to other spheres, such as change in life routine, and the probability of adverse effects or even sequela - especially the latter, in a disease that has subtle symptoms. Therefore, it is the quality of explanations and instructions provided by professionals to patients and/or caregivers that help to increase the chance of greater adherence to treatment and its success.

In diseases running a chronic course, in which treatment is prolonged or permanent, just like it is the case of congenital hypothyroidism, adherence to treatment is difficult to achieve.^6^ This factor was proved with the estimate that, among patients undergoing medication treatment, 50 to 65% of them do not adequately adhere to the proposed medical instructions,^27^ which leads to considering these patients as “difficult”, according to some studies.^6^ ^,^ ^8^ ^,^ ^23^ However, some of these studies did not consider other factors that could have an influence on treatment negligence on the part of patients, such as socioeconomic factors.

In the present study, a large portion of the caregivers evaluated had a poor level of knowledge about CH. This suggested that they only understood basic notions, essential for hormonal follow-up. However, they did not know how important it was to have good management of the way doses were administered and did not know about the deleterious effects of CH on the overall development of the child. This probably occurred due to lack of adequate guidance, considering that patients are submitted to an intense and specialized care routine in a single day, seen by different specialists in a short-time interval. This leads to professionals that focus on providing specialized care in their field of activity and fail to provide basic instructions to caregivers about the disease.

In developed countries, adherence to treatment for chronic diseases is estimated to achieve 50% of the population. In developing countries, this rate is much lower. This is partly due to the difficulty population has in accessing hospitals and care that would promote adherence to treatment. Therefore, developing policies and/or programs to encourage adherence to treatment is essential.^28^

Besides that, having a multidisciplinary team is of utmost importance, with the purpose of providing instructions and making sure that all doubts caregivers have are clarified. Moreover, creating educational workshops and public policies of informatization of basic health units and routine consultations is also crucial. This would guarantee more knowledge of the population about the disease, which could also develop efficient hormone follow-up, thereby improving adherence to treatment.

In addition, the low knowledge level of about the disease was related only to the educational level of participating caregivers, which was in disagreement with results showed by some studies.^6^ ^,^ ^23^ This divergence among studies may have occurred due to the difference in the number of participants in research, because they represented only one third of the number of caregivers who participated in the present study. The low level of instruction of caregivers consisted of a barrier to the process of health promotion: these individuals had greater difficulty in retaining new information, which frequently also involved terminology with which they were not familiar. These facts make clear the need for health professionals to develop a language closer to caregivers’ reality, in addition to monitor the level of information retained during each consultation.

In literature, successful adherence to treatment by patients and their families is possible when they have a good understanding of the costs and benefits associated with correct implementation of treatment is consensus.^26^ ^,^ ^29^ In addition, patients should follow the recommendations that make it easier for them to reconcile adhesion to treatment with their daily activities.

Although there is important evidence within the scope of health promotion, the present study design had limitations, and there were difficulties due to the fact that the questionnaire used as an instrumentfor evaluating knowledge of caregivers was not validated. However, it was evaluated in two pilot studies.

Despite these limitations, the aim of verifying knowledge of caregivers was attained. This allows the development of educational strategies to improve the service, which could be done in alliance with community health agents, given they are the mediators between health services and communities, thereby promoting an exchange of knowledge between individuals in the community. In addition, with a decentralization program in neonatal screening, children in a municipality would be followed up by a health professional, previously registered in the Secretary of Health. This would decrease the need for patients traveling from their cities to specialization centers and would improve adherence to treatment and access to information.

Nevertheless, further studies on this topic, with different research designs and validated questionnaires, must be developed to broaden the knowledge of individuals directly involved in the therapeutic process of patients with CH. The intention would be to help with the creation of new programs and public health policies designed for this population.

In conclusion, the results of the present study show the challenges related to adherence to medical treatment in congenital hypothyroidism, by patients and their families. Even though this treatment is relatively simple, easy to follow and of low cost, it may be considered an unsatisfactory metabolic control. According to the data presented, the main factors related to inefficient adherence to treatment of patients with CH are the educational level of caregivers and their knowledge level about the disease under treatment, which perhaps configures the main barrier to immediate and adequate follow-up of the affected children, probably demonstrating that well-instructed caregivers with essential information about the disease and its therapeutic processes present higher chances of adherence to treatment.
